# Metabolic Parameters as Biomarkers of Response to Immunotherapy and Prognosis in Non-Small Cell Lung Cancer (NSCLC): A Real World Experience

**DOI:** 10.3390/cancers13071634

**Published:** 2021-04-01

**Authors:** Lavinia Monaco, Maria Gemelli, Irene Gotuzzo, Matteo Bauckneht, Cinzia Crivellaro, Carlo Genova, Diego Cortinovis, Lodovica Zullo, Luca Carlofrancesco Ammoni, Davide Paolo Bernasconi, Giovanni Rossi, Silvia Morbelli, Luca Guerra

**Affiliations:** 1 School of Medicine and Surgery, University of Milano Bicocca, 20900 Monza, Italy; laviniamonaco92@gmail.com (L.M.); luca.guerra@unimib.it (L.G.); 2 Medical Oncology, ASST Monza, San Gerardo Hospital, 20900 Monza, Italy; maria.gemelli89@gmail.com (M.G.); d.cortinovis@asst-monza.it (D.C.); 3 IRCCS Ospedale Policlinico San Martino, 16132 Genoa, Italy; matteo.bauckneht@gmail.com (M.B.); silviadaniela.morbelli@hsanmartino.it (S.M.); 4 Nuclear Medicine Unit, Department of Health Sciences, University of Genoa, 16132 Genoa, Italy; 5 Nuclear Medicine, ASST Monza San Gerardo Hospital, 20900 Monza, Italy; cinzia.crivellaro@unimib.it; 6 UOC Clinica di Oncologia Medica, IRCCS Ospedale Policlinico San Martino, 16132 Genova, Italy; carlo.genova@hsanmartino.it; 7 Dipartimento di Medicina Interna e Specialità Mediche (DiMI), Facoltà di Medicina e Chirurgia, Università degli Studi di Genova, 16132 Genova, Italy; 8 UOC Oncologia Medica 2, IRCCS Ospedale Policlinico San Martino, 16132 Genova, Italy; lodozullo@gmail.com; 9 Medical Oncology, ASST Monza, San Gerardo Hospital, University of Brescia, 25123 Brescia, Italy; lucaammoni@gmail.com; 10 Bicocca Biostatistics Bioinformatics and Bioimaging Center—B4, School of Medicine and Surgery, University Milano Bicocca, 20128 Milano, Italy; davide.bernasconi@unimib.it; 11 Department of Medical, Surgical and Experimental Sciences, University of Sassari, 07100 Sassari, Italy; giovanni.rossi.1689@gmail.com; 12 UO Oncologia Medica, Ospedale Padre Antero Micone, 16153 Genova, Italy

**Keywords:** immunotherapy, NSCLC, PET/CT, response to therapy, metabolic tumor volume, quantification, OS, PFS

## Abstract

**Simple Summary:**

The advent of immune-checkpoint inhibitors (ICIs) has significantly changed the management of patients with non-small cell lung cancer (NSCLC). Although the assessment of PD-L1 expression remains the gold standard for the selection of cases for immunotherapy, previous experiments have investigated whether metabolic parameters from positron emission tomography (PET) scans could have a similar predictive role. In our retrospective study, we assessed if baseline fluorodeoxyglucose PET (FDG PET) can represent a further tool for selecting patients who are eligible for ICI therapy. Among the parameters tested, a surrogate of the entire tumor burden (metabolic tumor volume, MTV) demonstrated better performance in selecting patients with adequate disease control, as well as those with better overall and progression-free survival. These promising findings from a real-world experiment stress once again the pivotal role of PET scanning in the management of patients with NSCLC, requiring further validation on larger and prospective cohorts before its implementation in the stratification of patients for immunotherapy.

**Abstract:**

Immune-checkpoint inhibitors (ICIs) have been proven to have great efficacy in non-small cell lung cancer (NSCLC) as single agents or in combination therapy, being capable to induce deep and durable remission. However, severe adverse events may occur and about 40% of patients do not benefit from the treatment. Predictive factors of response to ICIs are needed in order to customize treatment. The aim of this study is to evaluate the correlation between quantitative positron emission tomography (PET) parameters defined before starting ICI therapy and responses to treatment and patient outcome. We retrospectively analyzed 92 NSCLC patients treated with nivolumab, pembrolizumab or atezolizumab. Basal PET/computed tomography (CT) scan parameters (whole-body metabolic tumor volume—wMTV, total lesion glycolysis—wTLG, higher standardized uptake volume maximum and mean—SUVmax and SUVmean) were calculated for each patient and correlated with outcomes. Patients who achieved disease control (complete response + partial response + stable disease) had significantly lower MTV median values than patients who had not (progressive disease) (77 vs. 160.2, *p* = 0.039). Furthermore, patients with MTV and TLG values lower than the median values had improved OS compared to patients with higher MTV and TLG (*p* = 0.03 and 0.05, respectively). No relation was found between the other parameters and outcome. In conclusion, baseline metabolic tumor burden, measured with MTV, might be an independent predictor of treatment response to ICI and a prognostic biomarker in NSCLC patients.

## 1. Introduction

Lung cancer remains the leading cause of tumor-related mortality, accounting for almost one-quarter of all cancer deaths in 2020 [1]. In recent years, the introduction of antibodies against programmed cell death protein-1 (PD-1) and its ligand (PDL1) have exhibited effective antitumor activity in non-small-cell lung cancer (NSCLC), with encouraging results [2,3,4,5,6]. However, the identification of patients who are likely to benefit from these therapies, as well as methods for evaluating the response to immune-checkpoint inhibitors (ICIs), remains challenging. Although the PD-L1 immunohistochemistry (IHC) expression level has been associated with better outcomes, the grade of expression alone is unsatisfactory as a sole biomarker to predict the efficacy of ICIs. Indeed, some patients with low/no PD-L1 (<50%) levels respond to these agents, whereas others with high PD-L1 expression (≥50%) do not [7]. At present, whether this is primarily due to artifacts related to limited tissue sampling or to under-appreciated facets of PD-L1 biology, including spatial and temporal heterogeneity, is still unclear [8]. Positron emission tomography/computed tomography (PET/CT) using radiolabeled glucose analog 2-[18F]-fluoro-2-deoxy-D-glucose (18F-FDG), is widely applied in oncology for non-invasive in vivo tumor characterization. PET/CT is an integral part of the clinical staging of patients with lung cancer [9] and plays an important role in the evaluation of therapeutic outcomes for clinical management. Unusual patterns of response on imaging, due to the novel mechanism of action of ICIs (T-cell activation) have been described and radiological images have demonstrated poor performance in this setting. Several studies have compared PET/CT to CT imaging in monitoring ICI treatment, with results suggesting an additional prognostic value of metabolic response assessments [10]. The role of pre-ICI 18F-FDG PET/CT scanning has been investigated in some monocentric retrospective studies, with different findings [11,12,13,14].

Some authors have described maximum standardized uptake value (SUVmax) as a potential predictive marker of response to ICIs [11,15], whereas others have not found any correlation [12]. Metabolic tumor volume (MTV) and total lesion glycolysis (TLG) appear to be the most promising quantitative indexes in predicting the therapy response [12,13,15]. A prognostic value for both MTV and TLG has been found in different kinds of malignancies [14,16,17,18], as demonstrated in previous studies assessing their values at baseline PET [19,20,21]. Even in NSCLC, both patients surgically treated at an early stage of disease [22] and advanced cases treated with chemotherapy [23,24] have demonstrated worse outcomes with high values of MTV and TLG. Moreover, Polverari et al. have shown the association of several radiomics features with progressive disease (PD) status, focusing on the primary lung lesion [12]. On this basis, in our bicentric study we aimed to assess the predictive role of pre-ICI 18F-FDG PET/CT quantitative parameters in the evaluation of therapy response and patient outcomes in advanced NSCLC patients.

## 2. Materials and Methods

### 2.1. Patients Selection and Ethical Aspects

This is a retrospective bicentric study conducted at two centers in Italy, the Ospedale San Gerardo ASST-Monza, Monza, Italy, and the IRCCS Ospedale Policlinico San Martino, Genova, Italy. Eligible patients had histological or cytological confirmed NSCLC at variable stages at the time of diagnosis and have been treated in any line of therapy with anti PD1 or PD-L1 medication (nivolumab, pembrolizumab or atezolizumab), regardless of the histological subtype, between 1 January 2015 and 1 March 2020. All patients underwent an FDG-PET/CT scan, performed within two months before starting immunotherapy. Previous surgery or radiotherapy and the presence of brain metastasis were allowed. A minimum follow-up of 3 months after treatment initiation and a first radiological evaluation was required for enrollment. Demographic (age, sex, smoke status), clinical (histologic subtypes, diagnosis, previous surgery/radiotherapy, prior lines and type of therapy) and molecular (EGFR, ALK, ROS1, BRAF, PD-L1, if available) data were also collected. Radiological assessment during treatment was performed as for good clinical practice every 8–12 weeks with CT-scan, FDG-PET/CT scan or both.

All patients’ data were anonymized by the local center at the time of enrollment and all procedures were performed in accordance with the ethical standards of the institutional and/or national research committee and with the 1964 Declaration of Helsinki and its later amendments or comparable ethical standards.

### 2.2. 18. F-FDG PET/CT Protocol

**Figure 1:**
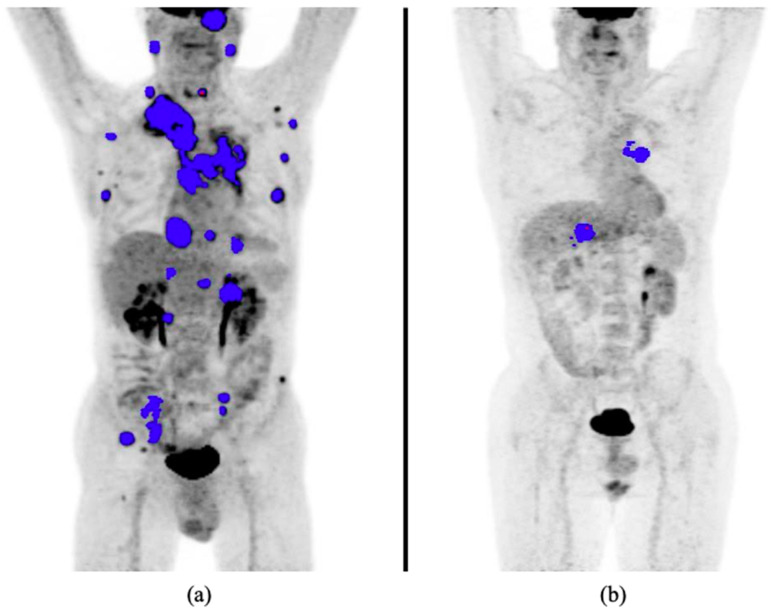
Pre-treatment MIP from 18F-FDG PET/CT in two different NSCLC patients with high (**a**) and low (**b**) MTV, respectively. MTV is calculated as the sum of all the MTVs from the countered lesions (blue voxels). 18F-FDG PET/CT, 18F-fluorodeoxyglucose positron emission tomography/computed tomography; MIP, maximum intensity projection; MTV, metabolic tumor volume.

Patients eligible for immunotherapy underwent a baseline 18F-FDG-PET/CT using a standard protocol (i.e., fasting for at least 6 h before the injection, blood glucose level < 150 mg/dL). PET/CT studies were performed with different PET/CT systems (Discovery 600, Discovery IQ and Discovery DMI, GE Healthcare, Milwaukee, WI, USA; Hirez-Biograph 16, Siemens Medical Solutions, USA) by acquiring a low-dose CT for attenuation-correction and anatomic localization of the FDG-avid lesions, followed by PET acquisition (1.5–2.5 min/bed depending on PET scanner). PET image reconstruction was obtained using ordered subset expectation maximization (OSEM) algorithms. PET, CT and fused PET/CT images were displayed on a dedicated workstation for reading, and abnormalities of metabolic activity were evaluated and classified as tumor-related or not by expert readers with more than 5 years of experience in clinical oncologic PET imaging. All metabolic tumor foci were then segmented using a threshold of 41% of SUVmax according to the European Association of Nuclear Medicine recommendations. MTV (mL) and TLG (g/mL/cm^3^) were automatically calculated by the software on the whole tumor foci (Figure 1), whereas the maximum (SUVmax) was evaluated for each metabolic active lesion; the highest SUVmax among the SUV of all the lesions was chosen for the analysis. The SUVpeak was then calculated by positioning a 10-mm-diameter circular region of interest (ROI) centered on the voxel of the lesion with the highest SUVmax among all the FDG-avid tumor foci. TLG was calculated by multiplying MTV by SUVmean.

### 2.3. Measures of Outcome

The aim of the present analysis was to assess the role of baseline quantitative PET parameters as prognostic and predictive response factors to immunotherapy in NSCLC patients. We investigated the association of SUVmax, SUVmean, SUVpeak, MTV and TLG with outcomes in terms of disease control rate (DCR) and with progression free survival (PFS) and overall survival (OS). Response to treatment was classified based on either clinical data or radiological criteria (Response Evaluation Criteria In Solid Tumors (RECIST) 1.1 or PET Response Criteria In Solid Tumors (PERCIST) 1.1 according to the imaging modality available for each patient) as complete remission (CR), partial response (PR), stable disease (SD) and progression of disease (PD). Patients with either SD, PR or CR were considered as “disease controlled”, whereas those with PD were assumed to be “non-controlled”. PFS was defined as the time from immunotherapy treatment initiation and disease progression or death. OS was calculated from the start of ICI therapy until death from any cause or censoring at the last time the patient was known to be alive.

### 2.4. Statistical Analysis

Categorical variables were described using absolute and relative frequencies; continuous variables were described using median and interquartile range (IQR). To assess the association with DCR, the distribution of each PET/CT parameter between disease control groups (CR-PR-SD vs. PD) was represented graphically by box-plots and was compared using the Mann–Whitney test. Overall PFS and OS over time were estimated using Kaplan–Meier curves. The association of PET/CT parameters with OS and PFS was investigated through Kaplan–Meier curves and the log-rank test (after dichotomization of PET/CT parameters using the median value as the cut-off) and by fitting univariate Cox models (considering PET/CT parameters as continuous variables). Finally, a multivariate Cox model was adopted to assess the association of SUVmean and MTV parameters, adjusting for gender, age and line of therapy. All the analyses were performed with R (version 4.0.3).

## 3. Results

### 3.1. Patient Characteristics

Ninety-two patients were enrolled in the study. Twenty-seven were female (29.3%). Median age was 70 years (interquartile range 62–75). Immunotherapy was used as the first line in 22.8% (21 patients) and as a second or further line in 71 (77.2%). Sixty patients (65.9%) were diagnosed with adenocarcinoma, 23 (25.3%) with squamous cell carcinoma (SCC) and eight (8.8%) with other histologic subtypes. Patient characteristics are listed in Table 1. Median PFS was 7.4 months (IQR 2.9–20.7). At the time of data cut-off 28 patients (30.4%) were alive. Median OS was 13.3 months (IQR 5.5–27.4). The medians of the metabolic parameters considered were: SUVmax 14.4 (IQR 11.0–18.8), SUVmean 4.9 (IQR 4.1–6.4), SUVpeak 9.2 (IQR 7.1–13.0), MTV 94.9 (IQR 31.3–240.2), TLG 542.6 (IQR 190.5–1314.9).

### 3.2. PET Parameters and Outcomes

**Figure 2:**
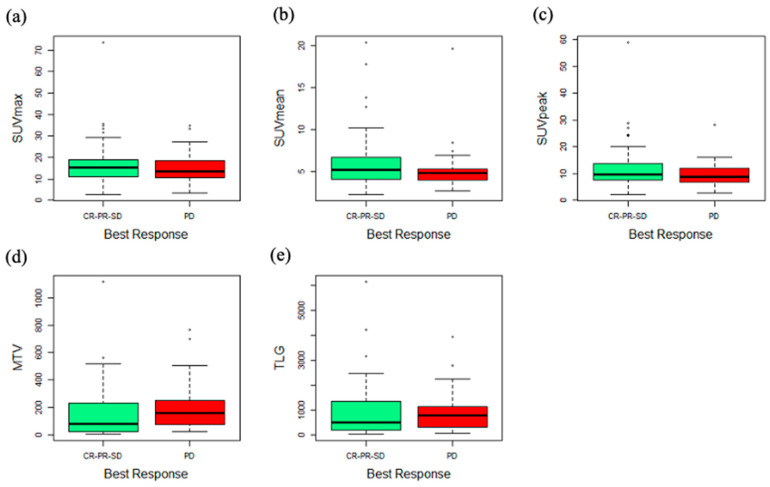
FDG-PET parameters evaluated comparing non-responders and responders to anti-PD-1 therapy. Box and whisker plot showing (**a**) standardized uptake volume maximum (SUVmax), (**b**) standardized uptake volume maximum (SUVmean), (**c**) standardized uptake volume peak (SUVpeak), (**d**) metabolic tumor volume (MTV), (**e**) total lesion glycolysis (TLG) of 92 NSCLC patients classified as responders (stable disease (SD), partial response (PR) or complete remission (CR), *n* = 61) and non-responders (progression of disease (PD), *n* = 31). The midline, box edges and outer bars indicate the median, first and third quartiles, and the upper and lower whiskers, respectively. Dots represent outliers.

Tumor response evaluation was available in all 92 patients. Two patients (2.2%) had CR, 26 (28.3%) PR, 33 (35.9%) SD and 31 (33.7%) PD, as best response. Patients who achieved disease control (CR + PR + SD) had significantly lower MTV median values than those who had not (PD) (77 vs. 160.2, *p* = 0.039, Figure 2).

Median +/− SD SUVmax, SUVmean, SUVpeak and TLG values in patients with disease control (CR, PR, SD) were 15.41 +/− 10.50, 5.21 +/− 3.45, 9.68 +/− 8.29 and 483.28 +/− 1099.22, respectively; the same parameters in patients without disease control (PD) were 13.45 +/− 7.48, 4.80 +/− 2.97, 8.82 +/− 4.71 and 767.40 +/− 876.65, respectively; all these parameters failed to show significant differences in the two groups of patients.

**Figure 3:**
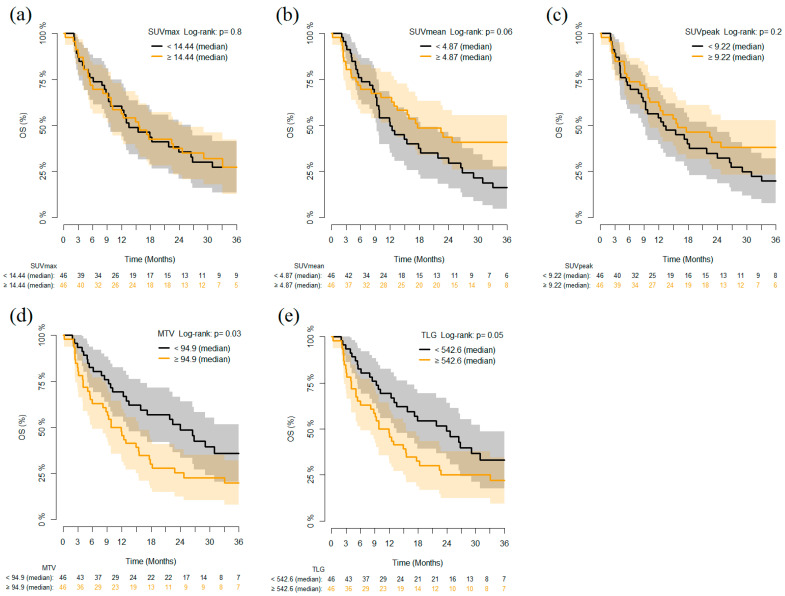
Kaplan–Meier curves of overall survival (OS) stratified according to dichotomized (on the median value) PET/CT parameters (**a**) SUVmax, (**b**) SUVmean, (**c**) SUVpeak, (**d**) MTV and (**e**) TLG in all patients (*n* = 92).

Considering outcomes, patients with MTV and TLG values lower than the median value (MTV 94.9 and TLG 542.6 respectively) had improved OS as compared to those with higher MTV and TLG (*p* = 0.03 and 0.05 respectively) (Figure 3).

In univariate analysis, SUVmean was found to be significantly associated with both PFS (Hazard Ratio [HR] 0.365 (95% confidence interval (CI) 0.150–0.890), *p* = 0.027) and OS (HR 0.261 (95% CI 0.084–0.808), *p* = 0.020), whereas MTV was correlated only with OS (HR 1.132 (95% CI 1.020–1.257), *p* = 0.020). Finally, MTV was the only parameter to be statistically associated with OS in the multivariate analysis as well (HR 1.221 (95% CI 1.078–2.124), *p* = 0.017) (Table 2).

## 4. Discussion

Different semiquantitative and quantitative parameters obtained from PET scans have been previously proposed as outcome predictors in various cancers, including NSCLC [25]. MTV, a surrogate of disease burden derived from the sum of all the MTVs from the contoured lesions, has been considered as a putative prognostic and predictive marker in patients treated with ICI. Although its prognostic role in baseline PET/CT has been extensively studied in other settings [26,27,28,29], its possible employment in patients treated with ICIs is controversial and data on its use in baseline assessment in melanoma [28] and NSCLC [11,12,13] are still scarce.

To the best of our knowledge, our retrospective study is among those with the largest cohorts for the evaluation of MTV in predicting therapy response in NSCLC. MTV values were significantly correlated with treatment response in terms of disease control, with higher MTV median values in patients with PD as compared to those with controlled disease (160.2 vs. 77; *p* = 0.039). A similar result was found in the retrospective study by Polverari et al. [12], in which patients with high MTV were more likely to be in the PD group, although their analysis of pre-therapy semi-quantitative values was limited to the primary lesion. A similar result has been described also by Evangelista et al. [11], where in a small cohort of 32 patients MTV was higher in non-responders, although not reaching statistical significance.

Moreover, in our population MTV was the only parameter that reached a significant association with OS in both univariate and multivariate analysis, showing that patients with MTV higher than median value (94.9 mL) had worse OS.

Chardin et al. [13] have recently defined baseline MTV as a strong predictive and prognostic biomarker in NSCLC patients treated with ICIs both in first and further lines of treatment; in their prospective study, higher MTV (>36.5 mL) at baseline PET was significantly associated with lower OS. Similarly, in two retrospective studies by Seban et al conducted on cohorts of 63 and 109 patients (88 considered eligible) with advanced NSCLC respectively [30,31], pre-treatment high total MTV (>75 cm^3^) combined with neutrophil to lymphocyte ratios correlated with poorer OS and PFS with no disease clinical benefit. Hashimoto et al. similarly demonstrated worse OS and PFS in patients treated with ICIs with high MTV (>5.0 mL) [32]. This variability in MTV cut-off values among different studies is likely mainly due to the diversity of various characteristics among populations enrolled.

Whether metabolic tumor burden expressed by MTV could predict the outcome of ICIs therapy remains unclear. Some authors have suggested that high hypoxia inducible factor-1 overexpressed in larger tumors may create an immunosuppressive environment in which Foxp3-regulatory T-cells (Tregs) have been reported to be positively associated with MTV [33,34,35]. We could thus speculate that a higher MTV could form a negative tumor microenvironment that could contribute to the resistance to ICI therapy. Further studies are needed to confirm and elucidate these results.

Results emerging from our study in accordance with the others mentioned above could be the proof of concept for a future role of pre-treatment MTV as a reliable, non-invasive prognostic biomarker in patients who are candidates for ICIs treatment, in order to find a robust optimal cut-off value for MTV in predicting the response to treatment and prognosis.

Other putative metabolic parameters that have been proposed in the evaluation of responses to ICI therapy are represented by different SUV values [36,37]. Although routinely used in the assessment of metabolic tumor response according to European Organization for Research and Treatment of Cancer (EORTC) and PERCIST criteria [36,37,38,39,40], their prognostic or predictive value in patients treated with ICIs is still controversial and the available data are contradictory. Some of the previous studies demonstrated a strong correlation between SUVmax and PD, although they were conducted on small cohorts [11,41]. Antithetical results were obtained by Takada et al.—in a cohort of 89 NSCLC patients treated with anti PD-1 therapy, pre-treatment SUVmax values higher than 11.6 correlated with better Objective Response Rate (ORR) (*p* = 0.0012), although this did not translate into a survival benefit [42]. This unexpected correlation could be explained by the possible presence of inflammatory cells (i.e., tumor-infiltrating lymphocytes (TILs) and tumor-associated macrophages), which are usually FDG-avid once activated. On the other hand, Polverari et al., in their study which focused on the evaluation of the metabolic state of the primary tumor lesion, failed to demonstrate significant correlation of SUVmax with any of the clinical outcomes examined [12]. Accordingly, considering the entire tumor load, no significant correlation was found among any SUV value (namely, SUVmean, SUVmax and SUVpeak) and the disease control indices (PD vs. DC). Although an association was noted between SUVmean and PFS/OS in univariate analysis, this has not been confirmed by multivariate analysis. The heterogeneity of these conflicting results could be explained by different reasons, such as the number of patients enrolled and evaluated, the possible interference of the metabolic state of the tumor microenvironment, the parameters used to evaluate the response to therapy and the tumor volumes considered (single primary lesion vs. total tumor load).

Our study has some limitations. First of all, the retrospective nature of the study may introduce some bias in the data collection. Moreover, PET analysis was performed in two experienced oncological centers but the reproducibility in MTV measurements has not been tested, although the same EANM guidelines for volume definition were shared and utilized. Finally, the population analyzed was quite heterogeneous due to the different histologic subtypes and lines of therapy used before starting ICI treatment. The correlation between SUV and PD-L1 expression was available only for a limited subset of cases. Despite these limitations, to our knowledge this is the largest population available in the literature and the first one to show a statistically significant correlation among SUVmean, MTV and outcomes in terms of DCR, PFS and OS.

## 5. Conclusions

Our data indicate that baseline metabolic tumor burden as measured based on MTV upon 18F-FDG-PET/CT in patients with NSCLC treated with ICIs was found to be an independent prognostic factor for OS and PFS. Furthermore, as a non-invasive, easily executable biomarker in clinical practice, MTV assessment could be a simple tool to predict patients’ responses to treatment, namely, DCR, and thus help clinicians to select patients who can really benefit from this innovative type of treatment. Validation with larger and prospective studies are needed to confirm our results.

## Figures and Tables

**Table 1:** Overall population (*n* = 92) characteristics.

Characteristics	Value
Age	Median 70 years old (range 61–75)
Sex	
Male	65 (70.7%)
Female	27 (29.3%)
Smoking status	
Non-smoker	10 (10.9%)
Ex-smoker	45 (48.9%)
Current smoker	32 (34.8%)
Not known	5 (5.4%)
Stage at first diagnosis	
IA	2 (2.2%)
IB	3 (3.3%)
IIA	2 (2.2%)
IIB	5 (5.4%)
IIIA	10 (10.9%)
IIIB	10 (10.9%)
IV	56 (60.9%)
Not known	4 (4.2%)
Histological variant	
Adenocarcinoma	60 (65.2%)
Squamous cell carcinoma	23 (25.3%)
NOS	8 (8.8%)
Not known	1 (1.1%)
Previous lung surgery	
No	65 (70.7%)
Yes	25 (27.2%)
Not known	2 (2.1%)
Immunotherapy	
First line	21 (22.8%)
Second line	39 (42.4%)
Third line	19 (20.7%)
Fourth line	13 (14.1%)
Molecule	
Atezolizumab + Bevacizumab	1 (1.1%)
Nivolumab	69 (75.0%)
Pembrolizumab	22 (23.9%)

**Table 2:** Multivariate Cox model on the association of SUVmean and MTV with PFS and OS, adjusted by some patient characteristics. In bold are highlighted the p-Values that reached statistical significance.

Variables	PFS		OS	
	HR (95%CI)	*p*-Value	HR (95%CI)	*p*-Value
SUVmean, per 10 units	0.423 (0.164–1.088)	0.074	0.337 (0.103–1.103)	0.072
MTV, per 100 units	1.139 (0.989–1.311)	0.07	1.221 (1.063–1.402)	0.005
Gender M vs. F	0.837 (0.506–1.384)	0.488	0.924 (0.533–1.600)	0.777
Age, per 10 years	1.287 (0.947–1.749)	0.108	1.523 (1.078–2.124)	0.017
First line vs. not first line	0.763 (0.390–1.496)	0.432	0.640 (0.278–1.873)	0.294

## Data Availability

The data presented in this study are available on request from the corresponding author. The data are not publicly available due to privacy reasons.
